# The MRX Complex Ensures NHEJ Fidelity through Multiple Pathways Including Xrs2-FHA–Dependent Tel1 Activation

**DOI:** 10.1371/journal.pgen.1005942

**Published:** 2016-03-18

**Authors:** Daichi Iwasaki, Kayoko Hayashihara, Hiroki Shima, Mika Higashide, Masahiro Terasawa, Susan M. Gasser, Miki Shinohara

**Affiliations:** 1 Department of Integrated Protein Functions, Institute for Protein Research, Osaka University, Suita, Osaka, Japan; 2 Department of Biological Science, Graduate School of Science, Osaka University, Suita, Osaka, Japan; 3 Research Institute for Radiation Biology and Medicine, Hiroshima University, Hiroshima, Japan; 4 Friedrich Miescher Institute for Biomedical Research, Basel, Switzerland; Duke University, UNITED STATES

## Abstract

Because DNA double-strand breaks (DSBs) are one of the most cytotoxic DNA lesions and often cause genomic instability, precise repair of DSBs is vital for the maintenance of genomic stability. Xrs2/Nbs1 is a multi-functional regulatory subunit of the Mre11-Rad50-Xrs2/Nbs1 (MRX/N) complex, and its function is critical for the primary step of DSB repair, whether by homologous recombination (HR) or non-homologous end joining. In human NBS1, mutations result truncation of the N-terminus region, which contains a forkhead-associated (FHA) domain, cause Nijmegen breakage syndrome. Here we show that the Xrs2 FHA domain of budding yeast is required both to suppress the imprecise repair of DSBs and to promote the robust activation of Tel1 in the DNA damage response pathway. The role of the Xrs2 FHA domain in Tel1 activation was independent of the Tel1-binding activity of the Xrs2 C terminus, which mediates Tel1 recruitment to DSB ends. Both the Xrs2 FHA domain and Tel1 were required for the timely removal of the Ku complex from DSB ends, which correlates with a reduced frequency of imprecise end-joining. Thus, the Xrs2 FHA domain and Tel1 kinase work in a coordinated manner to maintain DSB repair fidelity.

## Author Summary

Genomic DNA provides the essential blueprint for life, and therefore living organisms have several mechanisms for maintaining the stability of their own genomes. DNA double-strand breaks (DSBs) are one of the most severe forms of DNA damage, which, without precise repair, can provoke a loss of genetic information, leading to tumor formation. DSBs are repaired by two distinct pathways, homologous recombination (HR) and non-homologous end joining (NHEJ), which can be precise or imprecise. In addition, the DNA damage response (DDR) is essential in the cell to integrate multiple events that need to occur after damage: activation of DNA repair enzymes, selection of repair pathway and control of cell cycle progression, transcription, and so on. Here we show that different domains of Xrs2, a central DSB repair protein in budding yeast whose human ortholog, Nbs1, is linked to a human hereditary disorder with a high risk of cancer, is required not only for repair pathway choice but also for full activation of DDR. This result indicates that DSB repair and the DDR are coordinated at multiple levels to ensure precise repair and thus to maintain genomic integrity.

## Introduction

The DNA double-strand break (DSB) is one of the most severe types of DNA damage and is most often repaired by homologous recombination (HR) or canonical non-homologous end joining (C-NHEJ) which is known as precise NHEJ. There are, however, several other minor pathways for DSB repair, some of which generate serious rearrangements of DNA structure. It is thought that an incorrect choice among these repair pathways promotes genomic instability, which compromises biological activity and can with time, promote tumorigenesis in higher eukaryotes [1]. The Mre11-Rad50-Xrs2/Nbs1 (MRX/N) complex has many roles in the initial steps of DSB repair, whether by C-NHEJ or HR, and also in the recovery from stalled replication forks, in telomere maintenance, in meiotic recombination and in the Tel1/ATM-related DNA damage response (DDR) signaling [2–6]. Thus, MRX/N acts as an integrating hub of DDR pathways.

In budding yeast, *Saccharomyces cerevisiae*, repair by C-NHEJ requires several multi-subunit complexes, namely MRX, Yku70-Yku80 (Ku) and Dnl4-Lif1-Nej1 (DNA ligase IV). First, Ku binds to free double–stranded DNA (dsDNA) ends, without needing a specific DNA structure, and then is able to translocate to an internal region of the DNA molecule, including the single-stranded DNA (ssDNA) region [7, 8]. Then, C-NHEJ is completed by DNA ligase IV to rejoin the broken ends. To function effectively, these complexes rely on physical interactions between components, for example, Ku80 binds Dnl4 and Mre11 and Xrs2 binds Lif1 [9, 10]. Alternative non-homologous end joining (A-NHEJ), also known as microhomology-mediated end joining (MMEJ), is an auxiliary pathway for the repair of DSBs that occurs after end processing. MMEJ requires the MRX complex, Sae2, Tel1 and Rad1/Rad10/Slx4, but not Ku nor DNA ligase IV complexes [11, 12]. Although the process of MMEJ is quite similar to the single-strand annealing (SSA) reaction, MMEJ in yeast is genetically distinguishable from SSA by its requirement for Rad52, a protein that plays a key role in HR [11]. A further, minor pathway of NHEJ, often considered as a variant of C-NHEJ, is that of Ku-dependent imprecise end joining [13, 14]. This pathway can rejoin broken ends with or without microhomology after limited (<50 bases) resection [12]. It is unclear what molecular complexes or events distinguish C-NHEJ from the Ku-dependent imprecise–end joining reaction. Thus, MMEJ and Ku-dependent imprecise end joining are classified as imprecise NHEJ.

Mre11 has endo- and exonuclease activities, and Rad50 is a structural maintenance of chromosome (SMC)-like protein [15–18]. The Mre11-Rad50 sub-complex holds the two ends of a DSB together and facilitates their subsequent processing [19]. Mre11 and Rad50 are conserved from prokaryotes to mammals where they bind a third component called Nbs1 [20]. Xrs2 is the yeast ortholog of the Nbs1 subunit. Xrs2/Nbs1 is thus a eukaryote-specific multi-functional regulatory subunit of the MRX/N complex. The protein consists of a fork-head associated (FHA) domain, a pair of BRCA1 C terminus (BRCT) or BRCT-like domains, an Mre11-binding domain and a Tel1-binding domain [21–24]. The FHA domain is conserved in most of the orthologs in the N-terminal domain [21, 22, 25] and the motif generally has a well-known phospho-protein recognition function important for the DNA damage–related signaling pathway [26–28]. FHA domains are thus implicated in the recruitment of appropriate targets to sites of DNA damage through protein-protein interactions in the phosphorylation-transducing pathways, such as Rad53-Rad9 in budding yeast, Nbs1-Ctp1 in fission yeast and Nbs1-MDC1, or RNF8-MDC1, in humans [27–29].

In addition to the N-terminal FHA domain, the C-terminal region of Xrs2/Nbs1 harbors a Tel1/ATM-binding domain that is essential for Tel1/ATM recruitment to sites of DNA damage and to telomeres, as well as for its activity [22, 23, 30, 31]. In addition to Tel1/ATM, Mec1/ATR also has a role in the DDR reaction. Whereas Tel1/ATM recruitment depends on Xrs2/Nbs1, Mec1/ATR requires a replication protein A (RPA)-coated ssDNA stretch that results from Ddc2/ATRIP activity at the site of DNA damage [32]. Recent work suggests a role for RPA in MRX recruitment as well (Seeber A. *et al*., personal communication). In humans, truncation mutations of N-terminus region including FHA domain of Nbs1 have been identified as a causative factor for Nijmegen breakage syndrome (NBS), which confers a high risk of cancer and immunodeficiency and was, originally identified as an ataxia telangiectasia–like disorder [27, 33–36]. Consequently, cells in which the N-terminal region of NBS1 is truncated have abnormal cell cycle checkpoints, including the S-phase checkpoint, which is manifested as radio-resistant DNA synthesis [36, 37].

Here we report that dysfunction of the Xrs2 FHA domain, as with the loss of Tel1 kinase activity, leads to the accumulation of the Ku complex at DSB ends, which leads to an abnormal increase in imprecise end joining. Moreover, the Xrs2 FHA domain is required for robust activation of Tel1/ATM kinase at DSB ends both during mitosis and meiosis. Our findings reveal a genetic relationship between the Xrs2 FHA domain and Tel1 kinase activity in the maintenance of DSB repair fidelity and provide insights relevant to the human disease NBS.

## Results

### Mutations in the FHA domain cause an increase in imprecise end-joining within the same pathway as Tel1

**Fig 1:**
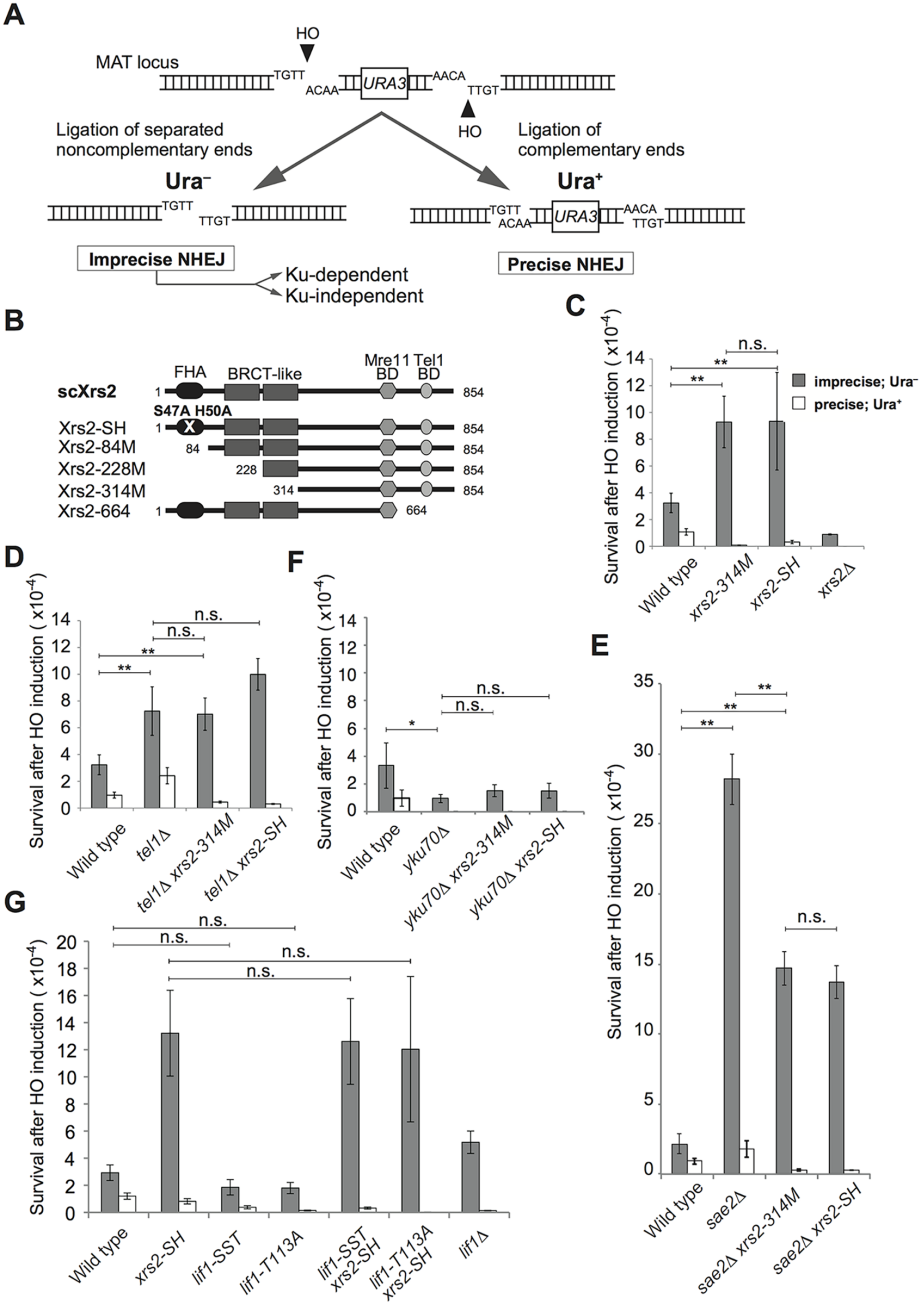
Imprecise end joining increased in cells with Xrs2 FHA domain mutations in a Tel1-dependent manner. A. System used to examine imprecise end joining. Two HO-cutting sites were introduced in opposite orientations within the *MAT* locus on chromosome III, that result in two kinds of DSB ends: non-complementary single-stranded over hangs (Ura^−^) and complementary overhangs (Ura^+^), which are repaired by imprecise end joining and precise end joining, respectively. B. Xrs2 protein domains for wild-type and mutant *Saccharomyces cerevisiae* (sc) proteins. Mre11 BD, Mre11-binding domain; Tel1 BD, Tel1-binding domain. C. Frequencies of Ura^−^ and Ura^+^ cells indicate imprecise end joining and precise end joining, respectively, in wild-type (SLY19), *xrs2-314M* (DIY002), *xrs2-SH* (DIY016) and *xrs2*Δ (DIY007) strains. D. End-joining frequencies in wild-type, *tel1*Δ (DIY129), *tel1*Δ *xrs2-314M* (DIY131) and *tel1*Δ *xrs2-SH* (DIY134) strains assessed as in C. E. End-joining frequencies in wild-type, *sae2*Δ (DIY059), *sae2*Δ *xrs2-314M* (DIY062) and *sae2*Δ *xrs2-SH* (DIY065) strains assessed as in C. F. End-joining frequencies in wild-type (SLY19), *yku70*Δ (DIY033), *yku70*Δ *xrs2-314M* (DIY051) and *yku70*Δ *xrs2-SH* (DIY048) strains assessed as in C. G. End-joining frequencies in wild-type, *xrs2-SH* (DIY134), *lif1-SST* (DIY027), *lif1-T113A* (DIY072), *lif1-SST xrs2-SH* (MSY5655), *lif1-T113A xrs2-SH* (MSY5652) and *lif1*Δ (KMY691) strains assessed as in C. The wild-type data in C, D and E are from a single experiment. Data are presented as the mean ± SD from three independent experiments. Significance was calculated with a Student’s *t*-test: **p<0.001; *p<0.05; n.s., not significant.

The FHA domain of Xrs2 is involved in NHEJ [9, 10, 22]. To learn more about the function of the FHA domain of Xrs2 in various NHEJ pathways, especially in imprecise end joining, we analyzed the effect of *xrs2* mutations on the repair of two HO endonuclease–induced non-complementary DSBs at the *MAT* locus in budding yeast [11]. In this system, two HO cleavage sites with opposite orientations were inserted on either side of *URA3* to allow repair by both Ku-dependent and Ku-independent pathways [14] (Fig 1A). The survival rate of Ura^−^ prototrophs corresponds to the frequency of repair of non-complementary DSB ends by imprecise end joining, which includes both MMEJ and Ku-dependent imprecise NHEJ (Fig 1A)[11, 12]. In contrast, the survival rate of Ura^+^ prototrophs, which are caused by re-ligation of two HO-induced complementary ends, corresponds to the frequency of precise NHEJ [11](Fig 1A). In an *xrs2*Δ mutant, as in *mre11*Δ and *rad50*Δ mutants [11], both imprecise end joining and precise NHEJ are compromised (Fig 1C).

Exonuclease defective mutations of *MRE11*, which encodes a protein that is in the same complex with Xrs2, show a completely different phenotype from that of *mre11*Δ in the various NHEJ pathways [14]. Thus, this assay is useful for revealing different functions within a given polypeptide. To determine the different roles played by Xrs2, we used two mutant alleles that compromise the FHA function of Xrs2: two point mutations in the FHA domain, *xrs2-SH* (S47A, H50A) and a truncation mutant that lacks 313 amino acids of the N-terminal domain, *xrs2-314M*, which eliminates both the FHA and the BRCT-like motifs (Fig 1B) [22]. These *xrs2* FHA mutants do not show any defects in MRX complex formation or, gamma ionizing radiation (γIR) sensitivity, but they do show a defect in NHEJ [10, 22]. We analyzed these mutants for their effects on various types of DSB repair and observed a significant 2.3- and 2.2-fold increase in the frequency of imprecise end joining in *xrs2-SH* and *xrs2-314M* mutants, respectively, relative to wild-type cells (Fig 1C). In contrast, the frequency of precise NHEJ, which was determined by the ratio of Ura^+^ prototrophs, was lower in these FHA domain mutants as compared with wild-type cells (Fig 1C), which is due to impaired interaction with Lif1 [10]. Total loss of Xrs2, in contrast, compromised both types of repair. This result indicated that the FHA domain of Xrs2 helps promote precise NHEJ and this or the FHA domain itself leads to the suppression of imprecise end-joining.

The *tel1*Δ and *sae2*Δ mutants were reported to show an increase in both precise NHEJ and imprecise end joining [12, 14], probably because of reduced resection at the break. Consistently, a truncation of *xrs2* (*xrs2-664*), which eliminates the Tel1-binding domain [22] (Fig 1B), results in an increase in the re-ligation of linearized plasmids *in vivo* [10]. Using our assay, we confirmed that the *tel1*Δ mutant had imprecise–end joining activity, which was 2.2-fold higher than that of wild-type cells (Fig 1D). Because the *tel1*Δ mutant was quite similar to these *xrs2* FHA mutants with respect to the frequency of imprecise end joining, we then examined the relationship between the two deficiencies by scoring imprecise end-joining in the double mutants. The frequencies of imprecise end-joining of *tel1Δ xrs2-314M* and *tel1Δ xrs2-SH* mutants were indistinguishable from that of the *tel1*Δ single mutant (Fig 1D), indicating that the increase in imprecise end joining in the *xrs2* FHA and *tel1*Δ mutants reflects the loss of a single pathway. Interestingly, however, the drop in precise NHEJ frequency in the *xrs2* FHA mutants was dominant over the increase observed in the *tel1*Δ mutant (Ura^+^, Fig 1D).

We observed a similar result upon loss of Sae2, which indirectly promotes Ku disassembly from DSBs through its activity in DSB end resection [38]. The imprecise–end joining frequency increased 10-fold over wild type, and the *sae2Δ xrs2* FHA double mutants showed a higher level of imprecise–end joining activity than did the *xrs2* FHA single mutants (Fig 1C and 1E). The effect on precise NHEJ (Ura^+^) frequencies showed a dominance similar to that of the *xrs2* mutants relative to *tel1*Δ (Fig 1D). However, the FHA domain of Xrs2 and Sae2 were not entirely epistatic to one another with respect to their effects on imprecise end joining (Fig 1E).

We further tested the effects of the *yku70*Δ mutation in these assays. *yku70*Δ was dominant over *xrs2* FHA mutations and suppressed the abnormal increase in imprecise end joining (Ura^−^) observed in the *xrs2* FHA mutants (Fig 1F). In addition, we confirmed that most of the residual imprecise end joining in the *yku70*Δ *xrs2* FHA double mutants was caused by the Ku-independent MMEJ pathway (S1 Fig). *yku* mutations are also dominant over *tel1*Δ mutations in imprecise end joining [14]. These results indicate that the increase in imprecise end joining in the *xrs2* FHA mutant is probably achieved by the Ku-dependent imprecise–end joining pathway, which also regulates events in the *tel1*Δ mutant.

In our previous study, we showed that the Xrs2 FHA domain functions in NHEJ through an interaction with Lif1, a component of DNA ligase IV in budding yeast [9, 10]. This was also confirmed in this assay as a reduced frequency of Ura+ prototrophs, as described above. Interestingly, the *xrs2* FHA mutation also suppressed precise NHEJ in the *tel1*Δ or *sae2*Δ background, which would most likely have been caused by an interaction defect with Lif1. To confirm this, we identified two Xrs2-interacting domains in Lif1 and constructed *lif1* mutations in each domain, named *lif1-SST* and -*T113A*, both of which lose the ability to interact with Xrs2 and compromise C-NHEJ [10]. We checked whether these *lif1* mutations allow an increase in imprecise end joining, as observed for the *xrs2* FHA-deficient mutant (Fig 1G). However, both *lif1-SST* and *lif1-T113A* mutants showed a slight decrease in the frequency of imprecise end joining, as compared with wild type, along with the expected drop in precise NHEJ (Fig 1G). In addition, both *lif1-SST xrs2–SH* and *lif1-T113A xrs2-SH* double mutants showed indistinguishable increases in imprecise end joining as compared with the *xrs2-SH* single mutant (Fig 1G). This confirms that the increase in imprecise end joining detected in the FHA-deficient mutants reflects its interaction with Tel1, rather than with Lif1. In contrast, lif1 mutations were dominant over the *xrs2-SH* mutation in the suppression of precise NHEJ. This indicates that precise NHEJ activity in the FHA-deficient mutants depends on the Lif1 interaction.

### A greater frequency of imprecise end joining with the FHA defect is caused by increased activity of the Ku-dependent pathway

**Fig 2:**
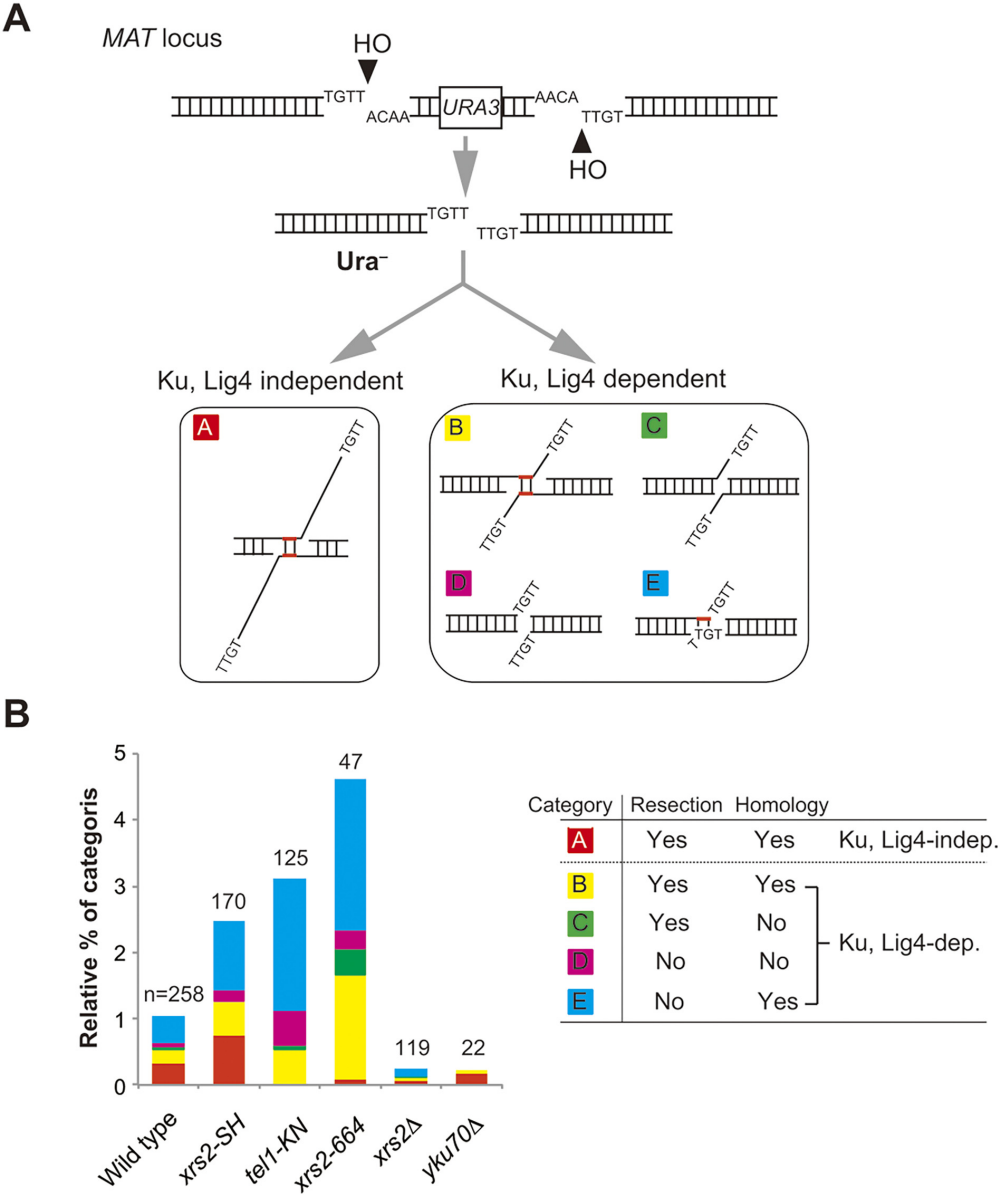
Distribution of DSB repair types produced by non-complementary DSB repair. A. The assay system and classification of the repair type depend on the junction analysis. Repaired products generated by survivors shown in Fig 1C and others were classified into five categories. Category A: repaired by annealing of microhomology with large asymmetric resection of up to 60 nucleotides in a Ku- and DNA ligase IV (Lig4)-independent manner. Category B: repaired by end joining mediated by annealing of microhomology with resection. Category C: repaired by end joining without microhomology-mediated annealing at resected ends. Category D: repaired by end joining without microhomology-mediated annealing and end resection. Category E: repaired by end joining with microhomology-mediated annealing without resection. Categories B–E are Ku and DNA ligase IV dependent. B. Distribution of each category of repair type in various strains. Left: Frequency of each category shown in A of the imprecise–end joining categories in wild type (SLY19), *xrs2-SH* (DIY016), *tel1-KN* (MSY4629), *xrs2-664* (MSY4835), *xrs2*Δ (DIY007) and *yku70*Δ (DIY033). Numerical values are shown in S3 Table. Right: Summary of classification of each category. n, number of samples analyzed for each strain.

We next characterized DSB-repair events observed after the induction of non-complementary DSBs. As shown previously [12], repair products can be classified into five categories (Fig 2A). To distinguish the categories, we determined the junctions of repaired products amplified from Ura^−^ prototrophs after induction of the non-complementary DSBs (Fig 1A). Products in category-A are produced by a Ku- and a DNA ligase IV–independent imprecise pathway [13, 39]. As expected, 95% of the products recovered in the *yku70* mutant belong to category A (Fig 2B and S1 and S2 Tables). This pathway is also called MMEJ in yeast [14] or A-NHEJ in mammalian cells [13]. In contrast, products in categories B–E are Ku dependent- and DNA ligase IV dependent [12] (S1 Fig and S2 Table). In addition, DSB repair products in categories B and C are associated with DSB end-resection, whereas those of categories B and E are mediated by microhomology-dependent annealing to repair non-complementary DSBs (Fig 2A and 2B, right).

We analyzed these repair events in *xrs2-SH*, *tel1* kinase-defective (*tel1-KN*), *xrs2-664*, *xrs2*Δ, and *yku70*Δ mutants for their effects in these end-joining events. First, we detected an increase in total frequencies for imprecise end joining in *tel1-KN* and *xrs2-664* (which truncates the Tel1-binding domain of Xrs2) mutants as well as in the *xrs2-SH* mutant (Fig 2B and S3 Table). It is remarkable that the increase in imprecise end joining in these mutants is associated with an increase in Ku- and DNA ligase IV–dependent repair, leading to products of categories B–E (Fig 2B, left). Only the *xrs2-SH* mutant maintained a high level of repair in category A, a Ku-independent pathway, as did wild-type cells (33.3 and 31.8%, respectively) (Fig 2B and S2 Table). In contrast, *tel1-KN* and *xrs2-664* mutants showed a substantial reduction in this category to 0.8% and 2.1%, respectively (S2 Table). This is consistent with a previous report showing that Tel1 function is essential for the Ku-independent MMEJ pathway [11]. In addition, category C products, which would be produced by simple re-ligation between processed ends, were not observed in the *xrs2-SH* mutant (<0.58%). This phenomenon is distinct from that of the *xrs2-664* and *xrs2*Δ mutants. Based on experiments with double mutants, the elevated level of imprecise end joining in *xrs2* FHA mutants was sensitive to the *yku70* mutation (Fig 1F, gray bar). Sequence analysis revealed that the *yku70*Δ *xrs2* FHA double mutant showed almost the same distribution for each category with the *yku*70Δ single mutant (S1 Fig). This also indicates that the *xrs2* FHA and *tel1* mutants promote an unusual Ku-dependent–imprecise end joining pathway (categories B–E).

### The Xrs2 FHA domain contributes to Ku removal from DSB ends

**Fig 3:**
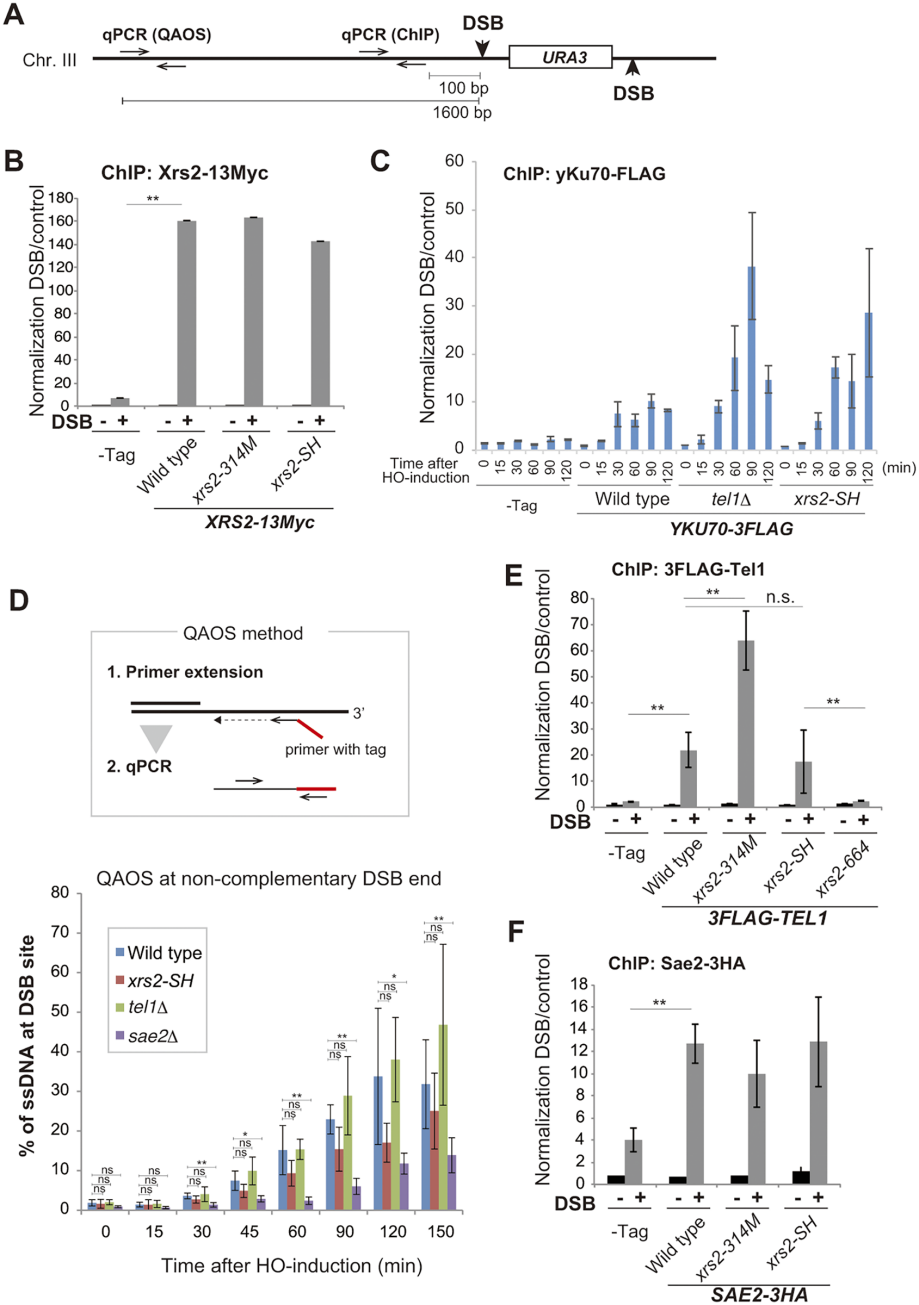
Recruitment of DDR proteins to non-complementary DSB ends. A. Construction of DSB sites at the MAT locus on chromosome III of strain SLY19 and position of the primer pair for qPCR for ChIP analysis and QAOS assay. B. The association of Xrs2 protein with DSBs in SLY19 (–Tag) or in its derivatives with *XRS2-13Myc* (wild type, DIY109; *xrs2-314M*, DIY116; *xrs2-SH*, DIY106). Error bars show the SD from three independent qPCR trials. C. Time course analysis of the association of yKu70 protein with DSBs in SLY19 (–Tag) or in its derivatives with *YKU70-3FLAG* (wild type, MSY4829; *tel1*Δ, DIY118; *xrs2-SH*, DIY120). Data are presented as the mean ± SD from four independent trials. Significance was calculated with a Student’s *t*-test: **p < 0.001; *p < 0.05; n.s., not significant. D. The QAOS method is shown (upper). ssDNA at DSB ends was detected by QAOS assay in wild type (SLY19), xrs2-SH (DIY016), *tel1*Δ (DIY129) and *sae2*Δ (DIY059) cells. Data are presented as the mean ± SD from three independent experiments. Significance was calculated with a Student’s *t*-test: **p < 0.001; *p < 0.05; n.s., not significant. E. The assembly of Tel1 protein on DSBs in SLY19 (–Tag) or in its derivatives with *3FLAG-TEL1* (wild type, MSY5505; *xrs2-314M*, MSY5539; *xrs2-SH*, MSY5486; *xrs2-664*, MSY5541). F. Association of Sae2 protein with DSBs in SLY19 (–Tag) or in its derivatives with *SAE2-3HA* (wild type, DIY142; *xrs2-314M*, DIY144; *xrs2-SH*, DIY146). In B and F or E, a ChIP assay was performed at the HO cutting site at 150 or 120 min, respectively, after HO induction (DSB +) or before induction (DSB–). Error bars in C, E and F show the SD from three or more independent experiments, each of which consisted of an average from three independent qPCR trials for each strain.

To assess which mechanisms were at work in the different mutants, we next analyzed the assembly of Xrs2, yKu70, Tel1 and Sae2 proteins on the HO-induced non-complementary DSB ends at the *MAT* locus on chromosome III by chromatin immunoprecipitation (ChIP). Quantitative real-time PCR (qPCR) was carried out with a primer pair that is 100 base pairs from the DSB site (Fig 3A). First, we examined assembly of mutant Xrs2 proteins at the DSB. We tagged wild-type Xrs2 and Xrs2–SH and Xrs2–314M mutant proteins with 13Myc epitopes at the C terminus, and confirmed normal recruitment of each mutant form following DSB induction (Fig 3B). Next, we examined assembly of the Ku complex at the DSB by using FLAG-tagged yKu70 in wild-type, *tel1*Δ, *xrs2-SH*, or *xrs2-314M* cells. Although FLAG-tagged yKu70 showed a moderate defect both in imprecise end joining and C-NHEJ, these levels of end-joining activity were substantially higher than in the *yku70*Δ mutant (S2A Fig). We observed a significant increase in yKu70 binding to the DSB ends in the *tel1*Δ, *xrs2-SH* and *xrs2-314M* mutants, compared with wild-type cells at 120 min after DSB-induction (Figs 3C and S2B). In addition, in the *xrs2-SH* and *tel1*Δ, Ku-binding signals were detected from 30 min after DSB induction as a same level with that in wild type, and then, they were gradually increased than wild type in accordance of time elapsed (Fig 3C). This indicates persistent binding of Ku at DSBs in the mutants. To determine whether a defect in the DSB resection indirectly affects the Ku removal from the DSB ends, we examined DSB resection in the *xrs2* FHA and *tel1*Δ mutants. We measured DSB end resection at 1600 base pairs from the DSB site (Fig 3A) by quantitative amplification of single-stranded DNA (QAOS) [40, 41] (Fig 3D). First, we confirmed that the *sae2*Δ mutant showed significantly reduced ssDNA production at the DSB ends, as reported [42]. Then we showed that *tel1*Δ had DSB resection with the same kinetics as did wild type. In contrast, the *xrs2-SH* mutant showed a slight decrease in ssDNA end production in the initial phase (15–120 min) but showed almost the same amount of resected DSB ends with wild type at 150 min, which is when accumulated yKu70 was observed at the ends (Fig 3C). This argues that Xrs2 works together with Tel1 to evict Ku from DSB ends during DSB resection.

As Xrs2 is involved in Tel1 recruitment to the DSB site though its C-terminal region [22] [23], we examined Tel1 binding at the DSB using FLAG-tagged Tel1. Interestingly, we detected Tel1 assembly at the DSB in *xrs2-SH* at wild-type levels and a significant increase in Tel1 binding in the *xrs2-314M* mutant cells (Fig 3E). This was distinct from the effect of the *xrs2-664* mutant, which lost Tel1 binding (Fig 3E), as previously reported [23]. This result indicates that the *xrs2* FHA mutation does not affect Tel1 recruitment to DSBs.

As it is known that Tel1/Mec1 phosphorylation is required for Sae2 function [43–45], we examined Sae2 recruitment using C terminally HA-tagged Sae2, but there was no difference between the wild-type recruitment and that in the *xrs2* FHA mutants (Fig 3F). This argues that FHA function of Xrs2 is not necessary for Sae2 binding at DSB sites. We conclude that Xrs2 FHA function does not affect the initial recruitment of Tel1 kinase to DSBs yet is responsible for removing Ku from the DSB ends. This was also observed in *tel1* mutant cells. We proposed that the Xrs2-FHA domain specifically supports Ku-removal by maintaining high Tel1 activity at DSB ends.

### The *xrs2* FHA mutation suppresses γIR sensitivity of *mec1*, and the lethality of *mec1* in the absence of the *sml1* mutation

**Fig 4:**
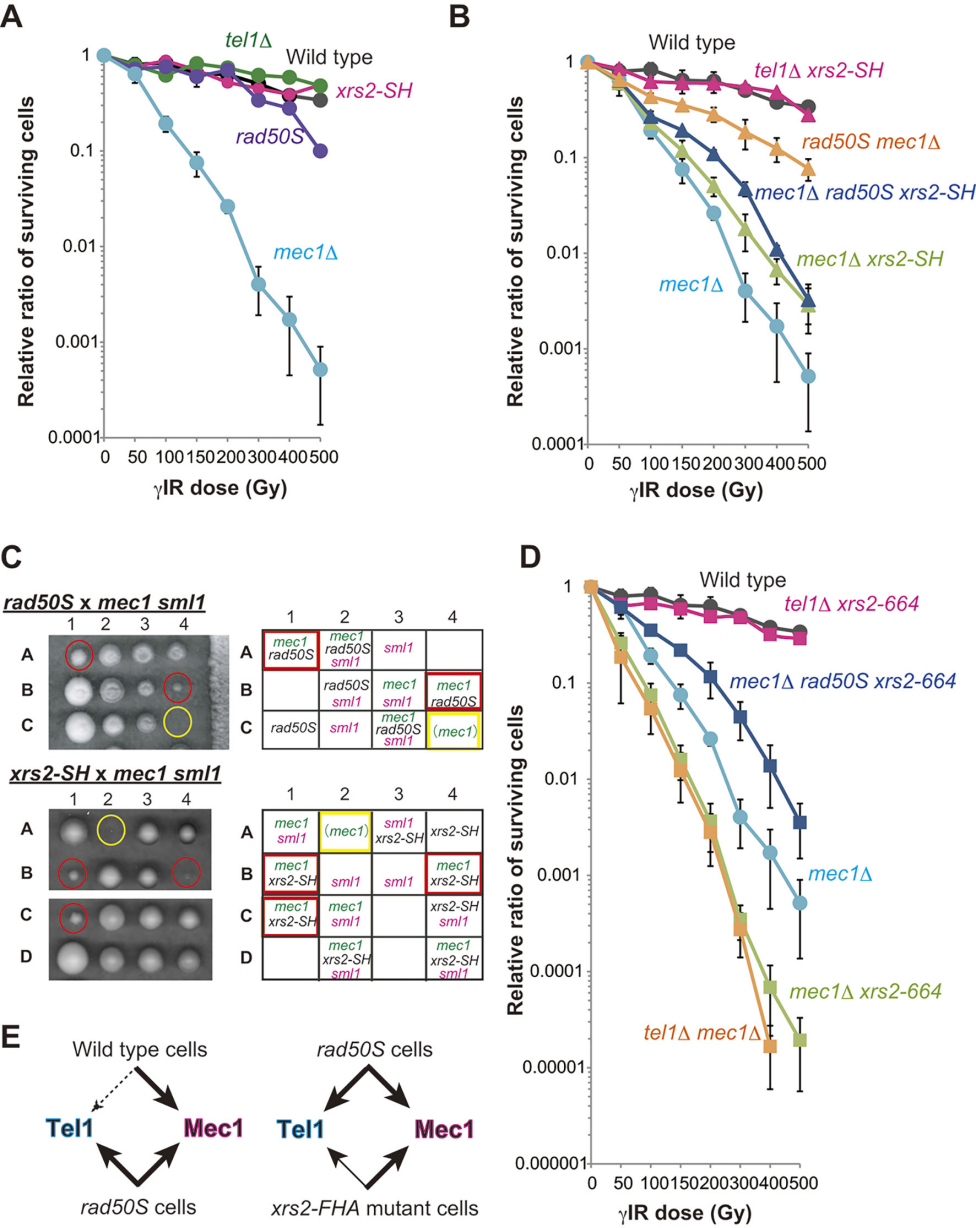
The FHA mutation partially up-regulates Tel1 and suppresses robust activation of Tel1 in the *rad50S* background. A. Survival of yeast cells after γIR of wild-type (W303-1A), *xrs2-SH* (MSY2199), *tel1*Δ (MSY2175), *rad50S* (MSY2203) and *mec1*Δ *sml1*Δ (shown as *mec1*Δ; MSY2211) cells. B. Survival after γIR of wild-type (W303-1A), *mec1*Δ *xrs2-SH* (MSY2331), *tel1*Δ *xrs2-SH* (MSY2319), *rad50S mec1*Δ (MSY2461), *mec1*Δ *rad50S xrs2-SH* (MSY2372) and *mec1*Δ (MSY2211) cells. C. Tetrad analysis of *rad50S mec1*Δ *sml1*Δ heterozygotes (MSY2205 × MSY2211; upper panel) and *xrs2-SH mec1*Δ *sml1*Δ heterozygotes (MSY2201 × MSY2211; lower panel). The genotypes of each dissected colony are shown. Red circles and rectangles indicate suppression of *mec1* lethality without the sml1 mutation. Yellow circles and rectangles indicate inviable colonies that were presumed to be *mec1*Δ cells based on the segregation pattern of the tetrads. D. Survival after γIR of wild-type (W303-1A), *mec1*Δ *xrs2-664* (MSY2309), *tel1*Δ *xrs2-664* (MSY2327), *rad50S mec1*Δ *xrs2-664* (MSY2356), *tel1*Δ *mec1*Δ (MSY2481) and *mec1*Δ (MSY2211) cells. The data shown in A, B, and D with the same label are based on the same set of experiments (that is wild type, *mec1*Δ). Error bars show the SD from three independent experiments. E. Conclusions from the irradiation analysis and tetrad analysis. The thickness of the arrow and the size of the arrowhead show the strength of the DDR in the indicated strains. Whereas Mec1 functions mainly in wild type, the *rad50S* mutation activates Tel1 activity (left). In contrast, an FHA mutation of *xrs2* only partially activates Tel1 activity in the *rad50S* background (right).

We demonstrated that the FHA domain of Xrs2 is related to the function of Tel1 in imprecise NHEJ suppression but not to its recruitment to DSB sites. To understand the function of the FHA domain of Xrs2 in a Tel1-dependent DDR pathway, we analyzed γIR sensitivity in the *xrs2* mutant cells. As reported previously [4], a *mec1*Δ mutant showed severe sensitivity to γIR because of defects in the DDR. In contrast, a Tel1-defective mutant did not show similar sensitivity (Fig 4A). This is because the Mec1-dependent pathway is the major pathway for survival in budding yeast after irradiation rather than the Tel1-dependent pathway [4]. *rad50S* and *sae2*Δ mutations, which have a defect in the initiation of DSB resection, can change the biased dependency on Mec1 by activating the Tel1 pathway through suppression of ssDNA production at broken ends [4, 42]. Thus, the *rad50S* mutation suppresses the radiation sensitivity of *mec1* mutant cells [4]. Consistently, the *mec1 rad50S* double mutant was 150-fold more resistant than *mec1*Δ alone to 500 Gy of γIR (Fig 4B).

We then examined whether the Xrs2 FHA domain plays a role in the DDR relative to γIR sensitivity. *xrs2-SH* is not sensitive to γIR [22], which we confirmed here; we also confirmed that *rad50S* mutant cells were only slightly sensitive to high doses of γIR (Fig 4A). Like *rad50S mec1*Δ, the *mec1*Δ *xrs2-SH* double mutant was 5.5-fold more resistant than *mec1*Δ alone to 500 Gy of γIR, indicating that the FHA mutation partially suppresses the DDR defect in *mec1*Δ. This suppression by *xrs2-SH* was less than that achieved with *rad50S*, however (Fig 2A and 2B). This suggests that *xrs2* FHA mutations may be able to change the biased dependency on Mec1 although activation of Tel1 might be incomplete.

*MEC1* is an essential gene in budding yeast, but *mec1*Δ cells grow in the presence of the *sml1* mutation [46]. Interestingly, *rad50S* also suppresses *mec1*Δ lethality presumably by activating the Tel1 pathway, even in the absence of *sml1*Δ [4] (Fig 4C, upper). Similarly, we found that the *xrs2-SH mec1*Δ double mutant was able to grow in the absence of the *sml1*Δ mutation, forming smaller but viable colonies (Fig 4C, lower). These results indicated that loss of the Xrs2 FHA domain suppresses γIR sensitivity in *mec1*Δ *sml1*Δ mutants and suppresses the lethality of the *mec1*Δ mutation. It remains to be tested whether this was through Tel1 recruitment or activation or through another pathway.

Tel1 activity *in vivo* depends largely on Xrs2, because of the physical interaction of Tel1 with a domain located in the C terminus of Xrs2 [23, 47]. Consistently, *xrs2-664* [22] did not show sensitivity to γIR even in a *tel1*Δ background (Fig 4D). Eliminating the Mec1 pathway in *xrs2-664* mutants, in contrast to *xrs2-664 tel1*Δ mutant cells, showed a severe loss of viability after irradiation, much like the *mec1*Δ *tel1*Δ double mutant (Fig 4D). In contrast to *xrs2-664*, the *xrs2-SH* mutation partially suppressed the *mec1*Δ defect, as described above (Fig 4B). Like the *mec1*Δ *rad50S xrs2-664* triple mutant, the *mec1*Δ *rad50S xrs2-SH* triple mutant was more sensitive to γIR than the *mec1*Δ *rad50S* double mutant (Fig 4B and 4D). Moreover, the *rad50S* mutation was not able to suppress the hypersensitivity of the *mec1*Δ *xrs2-SH* double mutant, especially at high doses of γIR (Fig 4B). This indicates that the *rad50S*-dependent suppression of *mec1*Δ hypersensitivity to γIR requires the FHA function of Xrs2, again acting most likely through Tel1 activation but not through the suppression of DSB processing in the *rad50S* mutation. We conclude that the Xrs2 FHA domain activates Tel1, although its function is distinct from that of the Tel1-binding domain of Xrs2.

### The FHA domain function of Xrs2 is not required for initial activation of Tel1 but is required for robust activation as well as its maintenance

**Fig 5:**
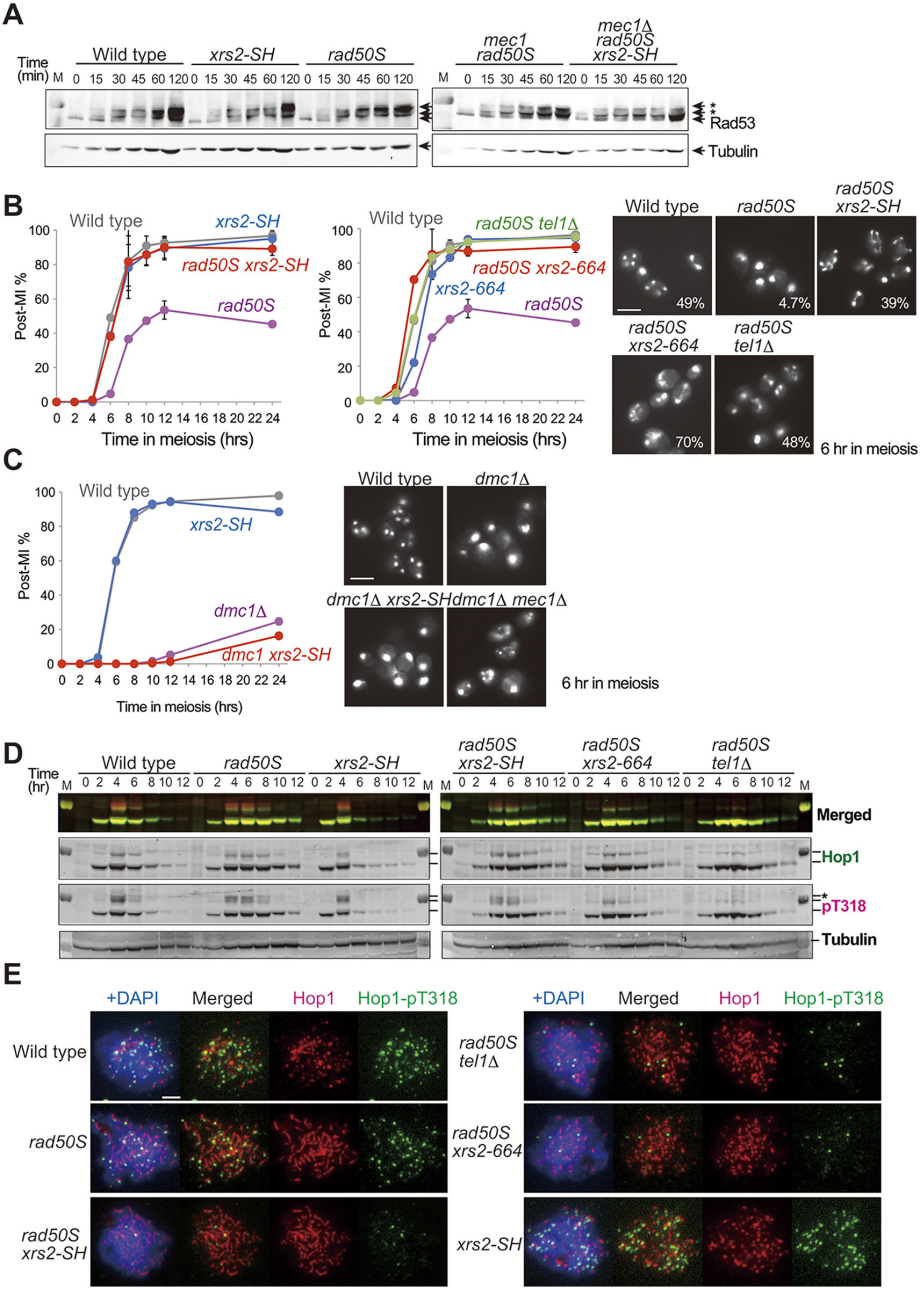
An FHA mutation suppresses Tel1 activity but not Mec1 activity during both mitosis and meiosis. A. Phosphorylation of Rad53 protein was analyzed at the indicated time points after phleomycin addition. Rad53 protein was detected by Western blotting by using anti-Rad53 in wild type (W303-1A), *xrs2-SH* (MSY2199), *rad50S* (MSY2203), *rad50S mec1*Δ (MSY2461) and *mec1*Δ *rad50S xrs2-SH* (MSY2372). Asterisks indicate phosphorylation signal of Rad53. Tubulin blots are shown as internal control for protein loading. B. Meiosis progression at the indicated time points after SPM transition in wild type (NKY1551), *rad50S* (MSY1758), *xrs2-SH* (MSY1867), *rad50S xrs2-SH* (MSY1844), *xrs2-664* (MSY2015), *rad50S xrs2-664* (MSY2106) and *rad50S tel1*Δ (MSY1952). The percentage of cells containing two or more nuclei per ascus (post-MI%) was plotted in the graphs. Images to the right show typical DAPI images after 6 hr of meiosis in each cell line, and the corresponding percentage of post-MI cells for each strain is shown. Scale bar indicates 2 μm. C. Meiosis progression in wild type (NKY1551), *xrs2-SH* (MSY1867), *dmc1*Δ (MSY2638) and *dmc1*Δ *xrs2-SH* (MSY4674). Typical DAPI micrographs after 6 hr of meiosis in each cell line, with addition of *mec1Δ dmc1*Δ as a control, are shown to the right. Scale bar indicates 2 μm. D. Phosphorylation of Hop1 at T318 (pT318) at the indicated time points after SPM transition was analyzed in wild type (NKY1551), *rad50S* (MSY1758), *xrs2-SH* (MSY1867), *rad50S xrs2-SH* (MSY1844), *xrs2-664* (MSY2015), *rad50S xrs2-664* (MSY2106) and *rad50S tel1*Δ (MSY1952). Hop1 protein was analyzed by western blot with anti-Hop1 (Hop1; green) or with anti-Hop1-pT318 antibody (pT318; magenta), and then each fluorescence signal was detected by laser scanner on the same membrane. Highly mobility shifted band was marked with asterisk. A merged image is shown on the top. Tubulin blots are shown as an internal control for protein loading. E. Localization of Hop1 (red) and Hop1-pT318 (green) on the meiotic nuclear spreads at 4 hr after SPM transition was analyzed in wild type (NKY1551), *rad50S* (MSY1758), *xrs2-SH* (MSY1867), *rad50S xrs2-SH* (MSY1844), *xrs2-664* (MSY2015), *rad50S xrs2-664* (MSY2106) and *rad50S tel1*Δ (MSY1952). Scale bar indicates 2 μm.

The Mec1-dependent pathway dominates the Tel1-dependent pathway in wild-type cells, but the Tel1 pathway will be equally activated in the *rad50S* mutant [4]. We hypothesized that this activation requires the Xrs2 FHA domain (Fig 4E) [40]. To test this hypothesis, we next examined DDR activity in the *xrs2* FHA mutant cells by monitoring Rad53 activation. Rad53 is a downstream mediator kinase of the yeast DDR pathway and is a phospho-target of Mec1 and Tel1 [48, 49]. We treated yeast haploid cells in vegetative growth with the DSB-inducing compound phleomycin and analyzed the Rad53 phosphorylation status by Western blotting at the indicated time points (Fig 5A). We detected step-wise accumulation of multiple slower-migrating signals, which correspond to different phosphorylated forms of Rad53, with robust phosphorylation achieved by 120 min in wild-type, *xrs2-SH* and *rad50S* cells (Fig 5A). We also detected an initial phosphorylation of Rad53 at 15 minutes after phleomycin addition and secondary phosphorylations events after 60 min in *mec1*Δ *rad50S* mutant cells. In the *mec1*Δ *rad50S xrs2-SH* triple mutant, the initial phosphorylation was also observed with the same timing as in the *mec1*Δ *rad50S* double mutant, but accumulation of secondary phosphorylation was delayed (Fig 5A). This indicates that the FHA domain of Xrs2 is not required for the initial step of Rad53 phosphorylation, although it is required for its robust activation in DDR signaling.

To clarify the FHA function in Tel1 activation, we analyzed the recombination checkpoint during meiosis in the *xrs2* FHA mutant. During meiosis, the *rad50S* mutation causes a complete block of DSB end resection because of the inability to remove covalent bonds between Spo11 and the DSB end; this leads to a Tel1-dependent delay in meiosis I entry [4, 50, 51]. In contrast, highly resected meiotic DSBs accumulate in *dmc1*Δ cells, provoking a Mec1-dependent arrest in prophase I [4, 52]. First, we examined the effects of the *xrs2* FHA mutation on progression meiosis I in the *rad50S* background. We observed that only 4.7% of the cells passed meiosis I in *rad50S* mutant cells after a 6-hr incubation in sporulation medium (SPM), which was substantially lower than that in wild type (49.1%, Fig 5B, left). The delayed entry into meiosis I in *rad50S* mutant cells was suppressed by the *tel1*Δ mutation, as reported previously [4], and a similar effect was observed with the *xrs2-664* mutation, reflecting the loss of Tel1-binding (Fig 5B, right). The *rad50S xrs2-SH* mutant cells also showed progression through meiosis I, with 39% having completed meiosis I after 6 hr in SPM, almost like wild-type cells (Fig 5B, left). This suppression was also observed in other FHA truncation mutants, namely *xrs2-314M*, *xrs2-228M* and *xrs2–84M* (S3A Fig), even with the different level of accumulation of un-resected DSBs in the *rad50S* background, which is caused by different amounts of Xrs2 protein [22]. Moreover, the *xrs2-664*/*xrs2-SH* heterozygotic diploid suppressed the delayed entry into meiosis I in the *rad50S* background (S3B Fig), indicating that the two alleles in *xrs2* do not complement each other through an intermolecular interaction. In contrast, the *xrs2-SH* mutation did not suppress prophase arrest in *dmc1****Δ*** (Fig 5C), which is Mec1-dependent, indicating that the function of the FHA domain of Xrs2 is not required for Mec1 activation.

A meiosis-specific axis component of the synaptonemal complex, Hop1, is phosphorylated at T318 by both Mec1 and Tel1 [53]. To monitor Tel1 activity, we thus analyzed Hop1 phosphorylation at Hop1-T318 by using an antibody specific for phospho-T318 in the *rad50S* background (Fig 5D and 5E). On Western blots, disappearance of Hop1 phosphorylation was delayed in *rad50S* (Fig 5D, pT318 asterisk), and the timing of T318 de-phosphorylation corresponded to the timing of the meiosis I transition (Fig 5B). The *xrs2-SH* mutation compromised Hop1-T318 phosphorylation when it was combined with *rad50S*, yet the level of phosphorylation was higher than that with the *tel1*Δ or *xrs2-664* mutations (Fig 5D, pT318 asterisk). Then we examined the localization of Hop1-pT318 on meiotic nuclear spreads after 4 hr in meiosis. In the *rad50S* single mutant, Hop1-T318 phosphorylation was observed as punctate foci on elongated Hop1 structures (Fig 5E). Although we observed normal Hop1 staining, only a few Hop1-T318 phosphorylation signals were observed in the *rad50S xrs2-SH* double mutant, similar to the staining in *rad50S tel1*Δ and *rad50S xrs2-664* cells. In contrast, in the *xrs2-SH* single mutant, Hop1-pT318 staining was indistinguishable from that of the wild-type cells (Fig 5E). This result suggests that FHA domain of Xrs2 is required especially for phosphorylation of chromatin bound Hop1 or maintains phosphorylated Hop1 at DSB sites. Finally, these results indicated that the FHA domain of Xrs2 is dispensable for the initial activation of Tel1 in the presence of DSB ends but it is required for its robust, prolonged activation, during both mitosis and meiosis.

## Discussion

We previously demonstrated that mutations in the FHA domain of *XRS2* cause defects in the rejoining of the ends of a linearized plasmid and in the repair of HO-induced (complementary) DSBs by binding the C-terminal region of Lif1 [10]. These events correspond to precise NHEJ *in vivo*. Here we showed that the Xrs2 FHA domain is involved in the suppression of imprecise end joining and, some extent, in the removal of Ku. Taken together, these results indicate that the fidelity of end-joining reactions is compromised by mutation of the Xrs2 FHA domain. Interestingly, mutation of the FHA domain leads to a defect in C-NHEJ [9, 10] but did not affect Ku-dependent imprecise end joining (Fig 2B). This indicates that Ku-dependent imprecise end joining is genetically distinguishable from C-NHEJ.

Nijmegen breakage syndrome in humans is caused by truncation of the N-terminal region, which contains FHA domain, of Nbs1, an ortholog of Xrs2. These patients exhibit a high risk of cancer, as well as immunodeficiency, which often results from a defect in the class switch recombination pathway [54]. In addition, activation of ATM, the human ortholog of Tel1, is required for AID/APE1 activation during class switch recombination [55]. Our work thus contributes to the understanding of this lethal human disease, which arises from alternative end-joining reactions. We have demonstrated that function of the Xrs2 FHA domain is needed for robust activation of Tel1 and for maintaining this activity during the DDR (Fig 5). However, the proper recruitment of Tel1 to a DSB site requires the Tel1-binding domain in the C terminus of Xrs2 [22, 23, 47]. We note that loss of the Xrs2 FHA domain does not impair Tel1 protein recruitment to an HO-induced DSB site, unlike the C-terminal truncation *xrs2-664* (Fig 3E). Interestingly, accumulation of Tel1 binding was observed in *xrs2-314M* but not in the *xrs2-SH* mutant. The *xrs2-314M* mutant lacks not only the FHA domain but also the BRCT-like domain (Fig 1B); it might thus be possible that the BRCT-like domain is involved in regulation of Tel1 stability at DSB ends. In addition, the *xrs2-314M* mutation produces a high amount of Xrs2 protein [22]. Although recruitment of excessive Xrs2 does not occur because Mre11 limits this step [22] (Fig 3B), free Xrs2 protein might affect Tel1 stability at the DSB ends. Moreover, the FHA domain of Xrs2 is required not only for activation, but also for suppression of Tel1 activity in the *mec1*Δ background (Fig 4C). Thus, Xrs2 is needed for regulation of Tel1 activation in multiple ways. The Xrs2-Tel1 interaction through the C-terminal domain of Xrs2 is not sufficient for robust activation and maintenance of Tel1 activity; the FHA domain of Xrs2 is required for a second step in Tel1 activation after recruitment.

FHA domains are phospho-protein recognition sites [26]. In budding yeast, the FHA domain of Xrs2 interacts with Lif1 and is involved in recruitment of DNA ligase IV complex through the interaction [10]. In the fission yeast, *Schizosaccharomyces pombe*, the FHA domain of Nbs1, an ortholog of Xrs2, interacts with Ctp1, an ortholog of Sae2 [56]. Similarly, phosphorylation at T90 of Sae2 is involved in its interaction with the Xrs2 FHA domain [57]. We analyzed the mutations of *sae2* phosphoacceptor-site (S4A Fig) [43, 58] with respect to the frequency of imprecise end joining. The resulting mutants showed phenotypes that were indistinguishable from that of the *sae2*Δ mutant, but were quite different from those of the *tel1*Δ and *xrs2* FHA mutants (S4B Fig, compare with Fig 1C and 1D). Thus Sae2 phosphorylation may create an interaction domain for Xrs2-FHA, although it probably also has other roles in repair. We note many of the human FHA domains that are associated with the BRCT domain, including that of Nbs1, recognize poly (ADP-ribose) and are involved in the DNA damage repair process [59]; poly-ADP ribosylation polymerases (PARPs), however, are absent in budding yeast [60].

The Xrs2 FHA domain was also required for robust activation of the Tel1 pathway during meiosis. We note that the initial Mec1 and Tel1 activation is shared between the mitotic DDR pathway and meiotic recombination checkpoint activation, yet the downstream signal transduction partners are quite different [61]. Xrs2 FHA function was not required for the Mec1-dependent pathway in meiosis (Fig 5C). Thus our results indicate that the Xrs2 FHA ligand may be a Tel1-specific target that is shared by the mitotic DDR and meiotic recombination checkpoint process. This could be Sae2, but there may be other candidates as well.

We showed that the *tel1*Δ mutation was epistatic to the *xrs2* FHA mutation with respect to an increase in imprecise end joining. In contrast, dysfunction of precise NHEJ in the FHA mutant results from a defect in the interaction of the DNA ligase IV complex with Lif1 [10]. Lif1 binding by Xrs2 FHA was not, however needed for suppression of imprecise end joining (Fig 1G). Collectively, these results argue that the Xrs2 FHA domain is multi-functional. Thus, in *xrs2* FHA mutant cells, addition to the defects in C-NHEJ through DNA ligase IV recruitment, compromised Tel1 activity would cause the abnormal increase of imprecise end joining.

We conclude that the enhancement of imprecise NHEJ in the *xrs2* FHA mutant is due to a partial defect in Tel1 function. Repair junction sequence analysis revealed that category A, which corresponds to Ku-independent A-NHEJ, was suppressed in *xrs2-664* mutant cells as in the *tel1-KN* cells, indicating that Tel1 recruitment to the DSB site and its kinase activity are essential for A-NHEJ at the DSB ends. The *xrs2* FHA mutant, however, only showed only a slight reduction in the frequency of A-NHEJ. This result suggests that robust activation of Tel1 is not essential for A-NHEJ formation.

**Fig 6:**
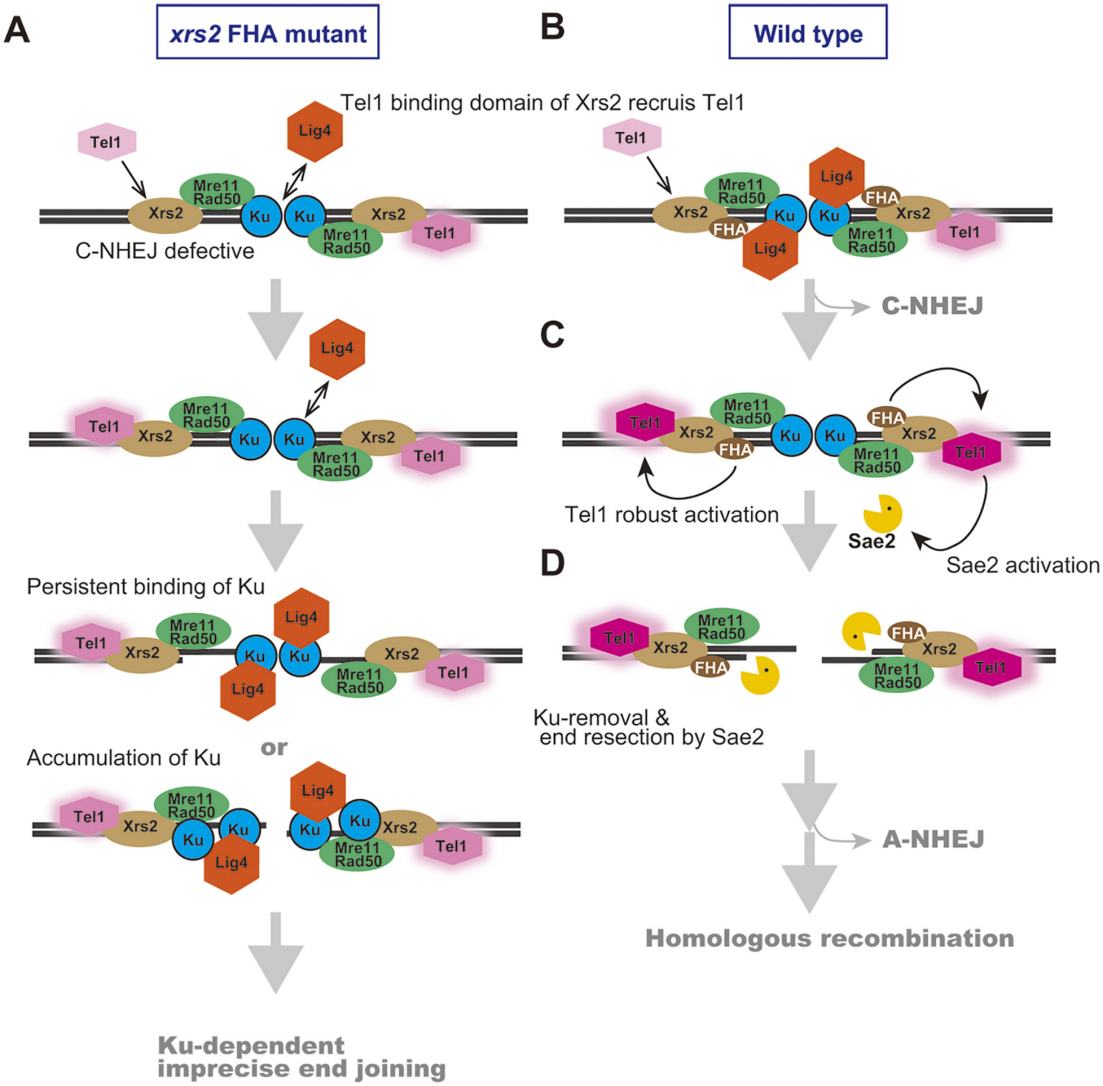
Proposed molecular mechanism for promoting imprecise end joining in the *xrs2* FHA mutants. A. DSB repair process in *xrs2* FHA mutants. Compromised Tel1 activity causes accumulation or persistent localization of Ku at processed DSB ends, which then often facilitates Ku-dependent imprecise end joining. B. Tel1 is recruited to DSB ends in a manner dependent on Xrs2 Tel1-binding domain. C. Then, robust activation of Tel1 is promoted in an Xrs2 FHA-dependent manner. The color change and halo effect of Tel1 indicates relative amounts of Tel1 activation. D. Phosphorylation of Sae2 or other unknown factors by Tel1 and/or Mec1 plays a role in DSB end resection as well as Ku removal to promote HR.

We found that the increase in imprecise end joining in the *xrs2* FHA mutant was caused by an increase in Ku-dependent products, corresponding to categories B–E (Fig 2B, S2 and S3 Tables). In addition, we showed that Ku accumulates at HO-induced DSB ends in the *xrs2* FHA mutants and also, more importantly, in the *tel1*Δ mutant, which did not show any defect in DSB end resection (Fig 3D). Therefore, the function of the Xrs2 FHA domain, acting through Tel1 activity, might promote Ku removal from DSB ends during end resection. Ku protein is first recruited to ds-DNA ends and possibly is then translocated to internal sites [7]. The abnormally high persistence of Ku protein or the accumulation of Ku protein at an inner region relative to processed DSB ends may activate incorrect end joining in the mutants through an interaction with Dnl4 [9] (Fig 6A). As previously noted, Tel1 activity is required not only for suppression of imprecise end joining but also for suppression of precise NHEJ [14](Fig 1D). In contrast, the *xrs2* FHA mutant had a defect in precise NHEJ because of a defect in the interaction between Xrs2 and Lif1. Ku removal thus may be an important function of Tel1 in the initial steps of DDR pathway choice. In vertebrate, another DDR sensor kinase, DNA-PKcs is involved in C-NHEJ and recruited to DSB site with Ku [62]. DNA-PKcs might take charge of the yeast Tel1 function specific to Ku regulation at DSB ends.

We envision the DSB repair process as follows: First, Tel1 is recruited to unresected DSB ends in an Xrs2 Tel1-binding domain–dependent manner (Fig 6B). Then, robust activation of Tel1 through the Xrs2 FHA function is promoted at DSB ends (Fig 6C). Phosphorylation of Sae2 by Tel1 and/or Mec1 plays an important role in DSB end resection as an initial step of HR [43, 44] (Fig 6D). Then, Sae2 and Mre11 promote subsequent Exo1 activity, which is required for extensive resection of DSB ends to facilitate efficient HR [63]. In contrast, Ku complexes compete with Exo1 at this step [63]. The robust activation of Tel1, which is dependent on the Xrs2 FHA domain, is needed to remove Ku from the processed ends to prevent Ku-mediated end-bridging as well as to allow efficient and extensive resection of DSB ends. If there is no resection needed, the FHA domain of Xrs2 can promote precise NHEJ by interacting with DNA ligase IV and then again it promotes Ku removal to ensure faithful DSB repair (Fig 6B). Given that NHEJ is the dominant mechanism of repair in mammalian cells, this latter pathway may be relevant for understanding how human NBS1 mutations in the FHA domain predispose cells to genomic instability and cancer.

## Materials and Methods

### Yeast strains

All yeast strains and their genotypes are shown in S1 Table. We used isogenic *Saccharomyces cerevisiae* W303-1A [64] derivatives for **γ**IR sensitivity determination, SLY19 [11] derivatives for the HO-induced non-complementary DSB rejoining assay and chromatin immunoprecipitation (ChIP) assay and the SK1 background NKY1551 [65] derivative for meiotic analysis.

### Preparation of antibodies

Rad53 and Hop1 antibodies were raised against purified recombinant proteins tagged with hexahistidine from *Escherichia coli*. Anti-Hop1-pT318 phosphospecific antibody was raised against synthesized polypeptide, ASIQPpTQFVSNC, and then, post-immune IgG was affinity purified by using this phosphopeptide and then titrated using a non-phosphorylated peptide, ASIQPTQFVSNC (custom-made by MBL Co., Ltd.).

### Noncomplementary DSB rejoining assay

End-joining activities to repair noncomplementary DSBs and repaired junction sequences were analyzed using SLY19 [11] and its derivatives as described [12]. Briefly, for each assay, a single colony was grown to log phase, and that culture was inoculated into YP-raffinose medium and incubated for 14 hr at 30°C to a concentration of 2.5 x 10^6^ cells/ml. Then, galactose was added to a final concentration of 2% (w/v). After an additional 2.5 hr incubation, cells were plated on YP-galactose. To quantify the total number of cells, cells were plated in parallel on YPAD plates after appropriate dilutions. For DNA sequencing analysis of repaired junctions, genomic DNA, purified from the cells grown on YP-galactose and shown to be Ura^−^, was analyzed as described [12].

### Determination of γIR sensitivity

Each strain was analyzed for γIR sensitivity as described [22]. A Shimadzu Isostron RTGS-21 was used with ^60^Co as the ionizing radiation source (Research Institute for Radiation Biology and Medicine, Hiroshima University).

### ChIP assay

The ChIP assay was performed as described [12] with minor modifications. Cells grown to mid-log phase in YP-raffinose medium were collected before galactose addition (DSB–) and at 150 min after galactose addition (DSB +), and were treated as described [12]. The following antibodies were used for immunoprecipitation: anti-DYKDDDDK tag (1E6, Wako) for yKu70-3FLAG and 3FLAG-Tel1, anti-HA (16B12, Covance) for Sae2-3HA and anti-Myc (MC045, Nacalai Tesque) for Xrs2-13Myc detection. qPCR was performed using the SYBR green system (SsoFast EvaGreen super mix and Chromo4, Bio-Rad) with primer sets at for *PHO5* on chromosome II (control) and for HO cutting sites (DSB) as follows: SLY19 DSB ChIP-f (5’-GGCCTTATAGAGTGTGGTCG-3’) and SLY19 DSB ChIP-r (5’-CAAAAGAGGCAAGTAGATAAGGG-3’). The specific recruitment of protein to HO-induced DSB ends was indicated as the relative ratio to the non-DSB locus (*PHO5*) control (DSB/control). The *y*-axis values (normalized DSB/control) are the relative ratios of the immunoprecipitation value (IP) to the input value (WCE; whole cell extract) as follows:

$$\text{Normalized DSB}/\text{control }=\;\left(\left({\text{IP}}_{\text{DSB}}/{\text{WCE}}_{\text{DSB}}\right)/\left({\text{IP}}_{\text{Control}}/{\text{WCE}}_{\text{Control}}\right)\right).$$

### QAOS assay

QAOS was performed at non-complementary DSB ends as described [40, 41]. Genomic DNA samples, which were purified at the indicated time points after HO induction, were used as the template. Primer extension reaction was carried out using a native genomic DNA sample and a heat-denatured DNA sample at 72°C with Taq DNA polymerase (Ex Taq, Takara Bio). qPCR was performed using the SYBR green system (Fast SYBR green master mix and Step one plus, Applied Biosystems) with primers for HO1 used in a previous study [41] after ExoSAP-IT (Affymetrix) treatment to remove primers from the previous reaction. The percent of ssDNA at the DSB site was calculated as follows:

$$\text{ssDNA }\%\;={\text{ QAOS}}_{\text{native}}/{\text{QAOS}}_{\text{denatured}}.$$

### Western blotting

Western blotting was performed as described [10]. Primary antibody binding was visualized with Alexa Fluor 680–labeled secondary antibodies (Molecular Probes) or IR dye 800–labeled secondary antibodies (Rockland) using an Odyssey infrared imaging system (LI-COR Biosciences). Antibodies used in these assays were anti-Rad53 (this study; rabbit, 1:1000), anti-Hop1 (this study, guinea pig, 1:1000), anti-Hop1-pT318 (this study; rabbit, 1:1000) and anti-α-tubulin (MCA77G; AbD Serotec).

### Meiotic analysis

Meiosis time course experiments were performed as described [66]. Meiotic progression was analyzed by counting the number of nuclei in each ascus under an epifluorescence microscope (Zeiss Axioskop 2) after staining with 4', 6-diamidino-2-phenylindole, dihydrochloride (DAPI). The frequencies of post–meiosis I cells containing two, three and four DAPI-stained bodies were determined. More than 200 nuclei were analyzed for each time point.

### Cytology

Immunostaining of yeast meiotic nuclear spreads was performed as described [10]. Stained samples were observed using an epifluorescence microscope (Zeiss Axioskop 2) with LED fluorescence light sources (X-Cite; Excelitas Technologies) and a 100× objective (Zeiss AxioPlan, NA1.4). Images were captured with a CCD camera (Retiga; Qimaging) and processed using IP lab (Silicon) and Photoshop (Adobe). Antibodies used in these assays were anti-Hop1 (1:1000) and anti-Hop1-pT318 (1:500).

## Supporting Information

S1 Fig Distribution of each category of repaired products in the *yku70*Δ background. Distribution of each category (%) of repaired products generated by survivors after non-complementary DSB induction in wild-type (SLY19), *yku70*Δ (DIY033), *yku70*Δ *xrs2-314M* (DIY051) and *yku70*Δ *xrs2-SH* (DIY048) strains. See Fig 2 for a description of the repair product categories.
(PDF)

S2 Fig Imprecise end joining and Ku binding at DSBs in the FLAG-tagged *YKU70* strain. A. Relative frequencies of survival rates of Ura^−^(gray bar, imprecise end joining) and Ura^+^ (white bar, precise end joining) cells in wild-type (SLY19), *yku70*Δ (DIY033) and *YKU70-3FLAG* (MSY4829) strains, which were used for ChIP analysis, are shown. Error bars show the SD from three or more independent experiments. B. The association of yKu70 protein with DSBs in SLY19 (–Tag) or in its derivatives with *YKU70-3FLAG* (wild type, MSY4829; *tel1*Δ, MSY4831; *xrs2-314M*, DIY118; *xrs2-SH*, DIY120) at 120 min after DSB induction.
(PDF)

S3 Fig Phenomenon of various *xrs2* mutations in the suppression of *rad50S*-dependent checkpoint pathway during meiosis. A. Meiosis progression at the indicated time points after transfer to SPM in wild type (NKY1551), *rad50S* (MSY1758), *xrs2–84M rad50S* (MSY1762), *xrs2-228M rad50S* (MSY1843) and *xrs2-314M rad50S* (MSY1992). The *xrs2-84M*, *-228M* and *-314M* mutations cause serial truncation of 83, 227 and 313 amino acids, respectively, at the N terminus of Xrs2 (Fig 1B). B. Meiosis progression was analyzed in wild type (NKY1551), *rad50S xrs2-SH* (MSY1844) and *rad50S xrs2-SH*/*xrs2-664* (MSY1817/2085) strains. The percentage of cells containing two or more nuclei per ascus (i.e., post-MI %) was plotted in A and B.
(PDF)

S4 Fig Imprecise end joining in the unphosphorylated mutants of *SAE2*. A. Tel1/Mec1-dependent phosphorylation sites in Sae2 protein and the amino acid positions in the phosphorylation mutants *sae2-5A*, *-3A* and *-2A*. B. Frequencies of survival of Ura^−^ and Ura^+^ cells indicate imprecise end joining and precise end joining, respectively, in wild-type (SLY19), *sae2-5A* (MTY1124), *sae2-3A* (MTY1125) and *sae2-2A* (MTY1127) strains.
(PDF)

S1 Table Yeast strains used in this study. (DOCX)

S2 Table Percentage of each category of repaired products after induction of non-complementary DSBs. (DOCX)

S3 Table Relative frequencies for each category of repaired products after induction of non-complementary DSBs. (DOCX)
