# Supplementary figures and images for: The MRX Complex Ensures NHEJ Fidelity through Multiple Pathways Including Xrs2-FHA–Dependent Tel1 Activation
Source: PLoS Genet. 2016 Mar 18;12(3):e1005942. doi: 10.1371/journal.pgen.1005942 (PMC4798412; doi:10.1371/journal.pgen.1005942)

Iwasaki D. et al., Figure S1

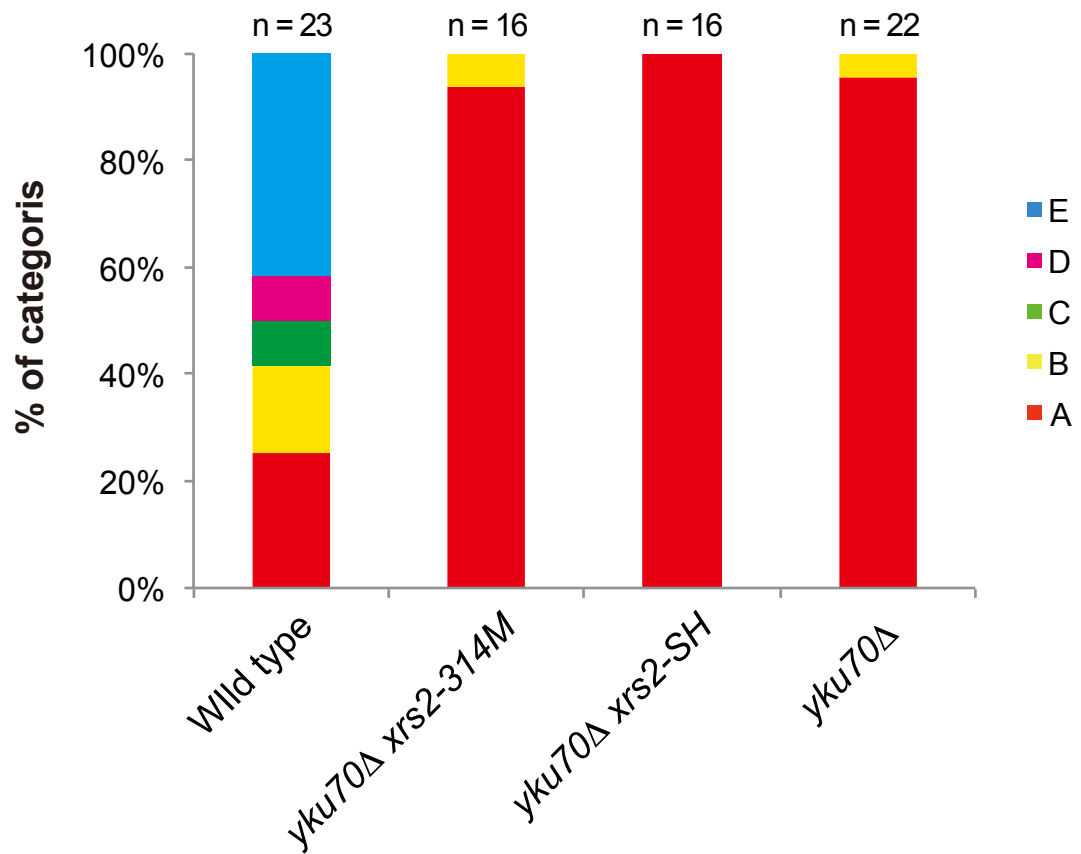

Supplement: S1 Fig — Distribution of each category (%) of repaired products generated by survivors after non-complementary DSB induction in wild-type (SLY19), yku70Δ (DIY033), yku70Δ xrs2-314M (DIY051) and yku70Δ xrs2-SH (DIY048) strains. See Fig 2 for a description of the repair product categories. (PDF) [file pgen.1005942.s001.pdf]

**A**

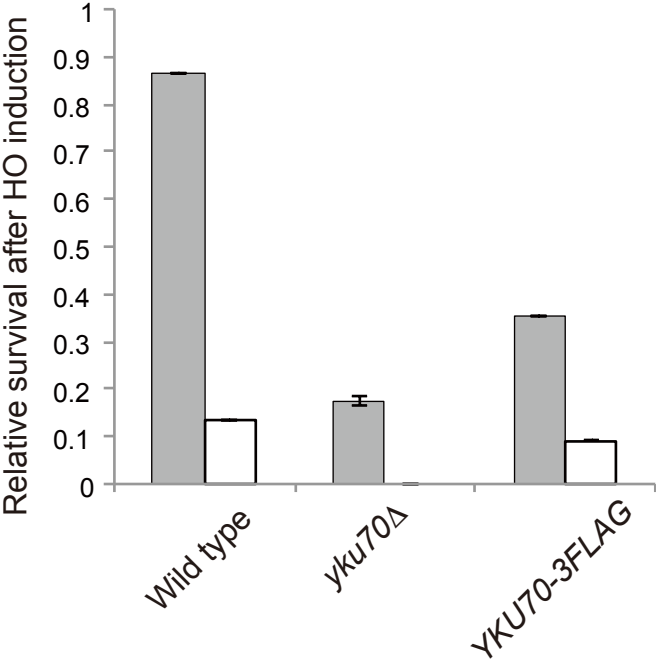

**B**

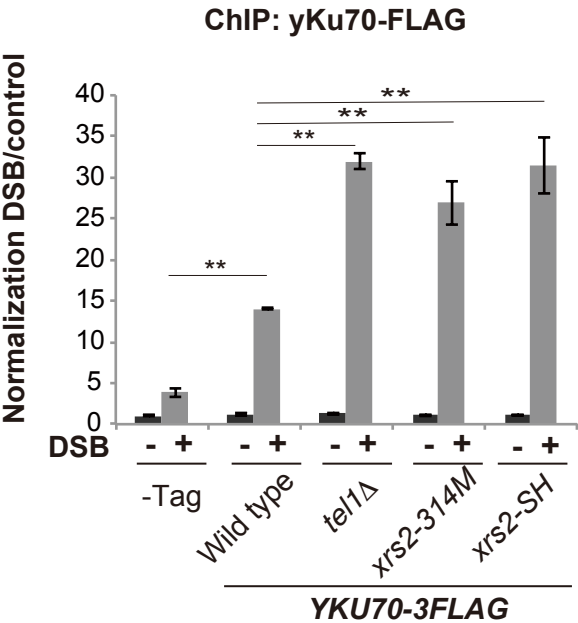

Supplement: S2 Fig — A. Relative frequencies of survival rates of Ura−(gray bar, imprecise end joining) and Ura+ (white bar, precise end joining) cells in wild-type (SLY19), yku70Δ (DIY033) and YKU70-3FLAG (MSY4829) strains, which were used for ChIP analysis, are shown. Error bars show the SD from three or more independent experiments. B. The association of yKu70 protein with DSBs in SLY19 (–Tag) or in its derivatives with YKU70-3FLAG (wild type, MSY4829; tel1Δ, MSY4831; xrs2-314M, DIY118; xrs2-SH, DIY120) at 120 min after DSB induction. (PDF) [file pgen.1005942.s002.pdf]

**A**

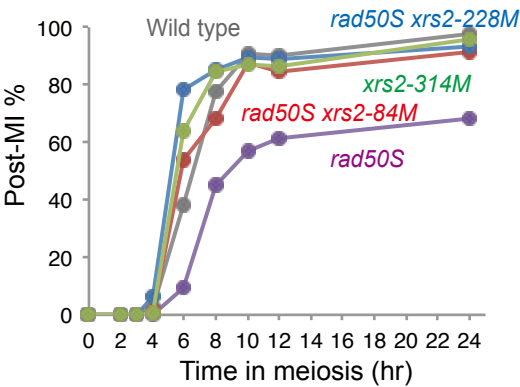

**B**

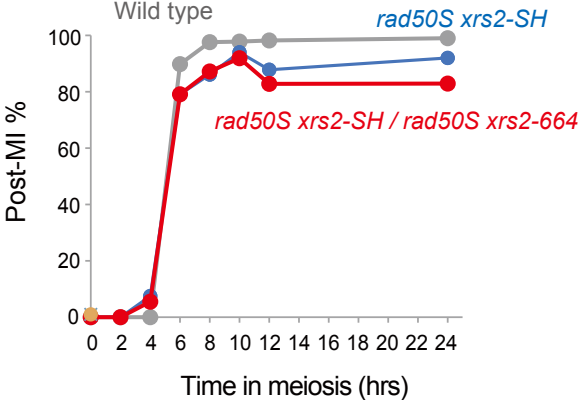

Supplement: S3 Fig — A. Meiosis progression at the indicated time points after transfer to SPM in wild type (NKY1551), rad50S (MSY1758), xrs2–84M rad50S (MSY1762), xrs2-228M rad50S (MSY1843) and xrs2-314M rad50S (MSY1992). The xrs2-84M, -228M and -314M mutations cause serial truncation of 83, 227 and 313 amino acids, respectively, at the N terminus of Xrs2 (Fig 1B). B. Meiosis progression was analyzed in wild type (NKY1551), rad50S xrs2-SH (MSY1844) and rad50S xrs2-SH/xrs2-664 (MSY1817/2085) strains. The percentage of cells containing two or more nuclei per ascus (i.e., post-MI %) was plotted in A and B. (PDF) [file pgen.1005942.s003.pdf]

**A**

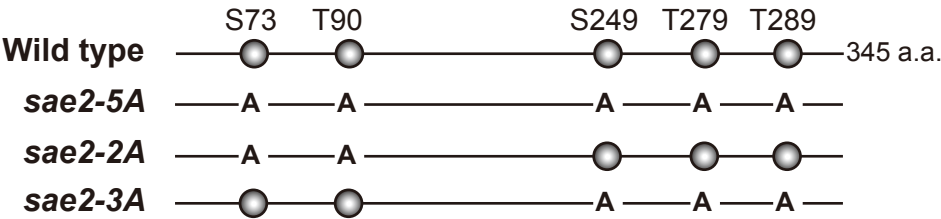

**B**

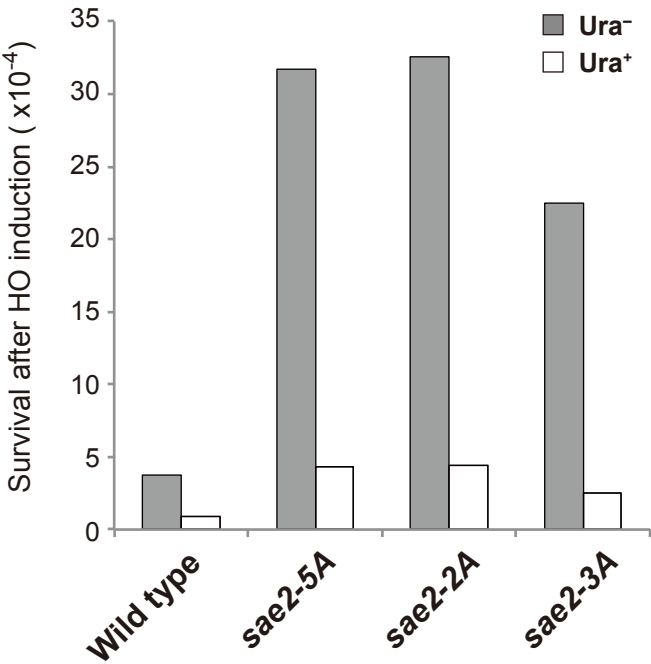

Supplement: S4 Fig — A. Tel1/Mec1-dependent phosphorylation sites in Sae2 protein and the amino acid positions in the phosphorylation mutants sae2-5A, -3A and -2A. B. Frequencies of survival of Ura− and Ura+ cells indicate imprecise end joining and precise end joining, respectively, in wild-type (SLY19), sae2-5A (MTY1124), sae2-3A (MTY1125) and sae2-2A (MTY1127) strains. (PDF) [file pgen.1005942.s004.pdf]
