# Supplementary material for: The MRX Complex Ensures NHEJ Fidelity through Multiple Pathways Including Xrs2-FHA–Dependent Tel1 Activation
Source: PLoS Genet. 2016 Mar 18;12(3):e1005942. doi: 10.1371/journal.pgen.1005942 (PMC4798412; doi:10.1371/journal.pgen.1005942)
Supplement: S1 Table — (DOCX) [file pgen.1005942.s005.docx]

**Table S1. Yeast strains used n this study**

| Strain | Genotype | Reference |
| --- | --- | --- |
| SLY19 | *MATα::URA3::HO hoΔ hmlΔ::ADE1 hmrΔ::ADE1 ade1-100 leu2-3,112 lys5 trp1::hisG ura3-52 ade3::GAL::HO* | [[1](#_ENREF_1)] |
| DIY002 | Derivative of SLY19 with *xrs2–314M* | This study |
| DIY016 | Derivative of SLY19 with *xrs2–SH* | This study |
| MSY4835 | Derivative of SLY19 with *xrs2–664* | This study |
| DIY007 | Derivative of SLY19 with *xrs2::KANMX6* | This study |
| DIY129 | Derivative of SLY19 with *tel1::TRP1* | This study |
| MSY4629 | Derivative of SLY19 with *tel1-KN* (N2612A, N2616A) | This study |
| DIY059 | Derivative of SLY19 with *sae2::hphMX4* | This study |
| KMY691 | Derivative of SLY19 with *lif1::KANMX6* | [[2](#_ENREF_2)] |
| DIY072 | Derivative of SLY19 with *lif1–T113A* | This study |
| DIY027 | Derivative of SLY19 with *lif1–SST (S383, S385, T387A)* | [[2](#_ENREF_2)] |
| MSY5652 | Derivative of SLY19 with *lif1–T113A xrs2-SH* | This study |
| MSY5655 | Derivative of SLY19 with *lif1–SST xrs2-SH* | This study |
| DIY033 | Derivative of SLY19 with *yku70::LEU2* | This study |
| DIY131 | Derivative of SLY19 with *xrs2–314M tel1::TRP1* | This study |
| DIY134 | Derivative of SLY19 with *xrs2–SH tel1::TRP1* | This study |
| DIY051 | Derivative of SLY19 with *xrs2–314M yku70::LEU2* | This study |
| DIY048 | Derivative of SLY19 with *xrs2–SH yku70::LEU2* | This study |
| DIY062 | Derivative of SLY19 with *sae2::hphMX4 xrs2–314M* | This study |
| DIY065 | Derivative of SLY19 with *sae2::hphMX4 xrs2–SH* | This study |
| DIY109 | Derivative of SLY19 with *XRS2-13myc::TRP1* | This study |
| DIY116 | Derivative of SLY19 with *xrs2–314M-13myc::TRP1* | This study |
| DIY106 | Derivative of SLY19 with *xrs2–SH-13myc::TRP1* | This study |
| MSY4829 | Derivative of SLY19 with *YKU70-3FLAG::KANMX4* | This study |
| DIY118 | Derivative of SLY19 with *xrs2–314M YKU70-3FLAG::KANMX4* | This study |
| DIY120 | Derivative of SLY19 with *xrs2–SH YKU70-3FLAG::KANMX4* | This study |
| MSY4831 | Derivative of SLY19 with *tel1::TRP1 YKU70-3FLAG::KANMX4* | This study |
| MSY5505 | Derivative of SLY19 with *3FLAG-TEL1* | This study |
| MSY5539 | Derivative of SLY19 with *3FLAG-TEL1 xrs2–314M* | This study |
| MSY5486 | Derivative of SLY19 with *3FLAG-TEL1 xrs2–SH* | This study |
| MSY5541 | Derivative of SLY19 with *3FLAG-TEL1 xrs2–664* | This study |
| DIY142 | Derivative of SLY19 with *SAE2-3HA::KANMX4* | This study |
| DIY144 | Derivative of SLY19 with *xrs2–314M SAE2-3HA::KANMX4* | This study |
| DIY146 | Derivative of SLY19 with *xrs2–SH SAE2-3HA::KANMX4* | This study |
| MTY1124 | Derivative of SLY19 with *sae2-5A* | This study |
| MTY1125 | Derivative of SLY19 with *sae2-3A* | This study |
| MTY1127 | Derivative of SLY19 with *sae2-2A* | This study |
| W303-1A | *MATa ade2-1 can1-100 his3-11,15 leu2-3,112 trp1-1 ura3-1* | [[3](#_ENREF_3)] |
| MSY2199 | Derivative of W303–1A with *xrs2–SH* | [[4](#_ENREF_4)] |
| MSY2273 | Derivative of W303–1A with *xrs2–664* | [[4](#_ENREF_4)] |
| MSY2175 | Derivative of W303–1A with *tel1::TRP1* | This study |
| MSY2203 | Derivative of W303–1A with *rad50S* | This study |
| MSY2211 | Derivative of W303–1A with *mec1::LEU2 sml1::KANMX6* | This study |
| MSY2481 | Derivative of W303–1A with *mec1::LEU2 sml1::KANMX6 tel1::TRP1* | This study |
| MSY2331 | Derivative of W303–1A with *xrs2–SH mec1::LEU2 sml1::KANMX6* | This study |
| MSY2319 | Derivative of W303–1A with *xrs2–SH tel1::TRP1* | This study |
| MSY2461 | Derivative of W303–1A with *rad50S::URA3 mec1::LEU2 sml1::KANMX6* | This study |
| MSY2372 | Derivative of W303–1A with *xrs2–SH rad50S::URA3 mec1::LEU2 sml1::KANMX6* | This study |
| MSY2309 | Derivative of W303–1A with *xrs2–664 mec1::LEU2 sml1::KANMX6* | This study |
| MSY2327 | Derivative of W303–1A with *xrs2–664 tel1::TRP1* | This study |
| MSY2297 | Derivative of W303–1A with *xrs2–664 rad50S::URA3* | This study |
| MSY2356 | Derivative of W303–1A with *xrs2–664 rad50S::URA3 mec1::LEU2 sml1::KANMX6* | This study |
| W303-1B | *MAT alpha ade2-1 can1-100 his3-11,15 leu2-3,112 trp1-1 ura3-1* | [[3](#_ENREF_3)] |
| MSY2205 | Derivative of W303–1B with *rad50S::URA3* | This study |
| MSY2201 | Derivative of W303–1B with *xrs2–SH* | This study |
| NKY1551 | *MATa/alpha ho::LYS2/ho::LYS2 ura3/ura3 leu2::hisG/leu2::hisG lys2/lys2 his4X-LEU2::BamHI-URA3/his4B-LEU2 arg4-Nsp/arg4-Bgl* | [[5](#_ENREF_5)] |
| MSY1867 | Derivative of NKY1551 with *xrs2–SH* | [[6](#_ENREF_6)] |
| MSY1992 | Derivative of NKY1551 with *xrs2–314M* | [[6](#_ENREF_6)] |
| MSY2015 | Derivative of NKY1551 with *xrs2–664* | [[6](#_ENREF_6)] |
| MSY1758 | Derivative of NKY1551 with *rad50S::URA3* | [[6](#_ENREF_6)] |
| MSY1844 | Derivative of NKY1551 with *rad50S::URA3 xrs2–SH* | This study |
| MSY2106 | Derivative of NKY1551 with *rad50S::URA3 xrs2–664* | This study |
| MSY1762 | Derivative of NKY1551 with *rad50S::URA3 xrs2–84M* | [[6](#_ENREF_6)] |
| MSY1843 | Derivative of NKY1551 with *rad50S::URA3 xrs2–228M* | [[6](#_ENREF_6)] |
| MSY1992 | Derivative of NKY1551 with *rad50S::URA3 xrs2–314M* | [[6](#_ENREF_6)] |
| MSY1952 | Derivative of NKY1551 with *rad50S::URA3 tel1::TRP1* | This study |
| MSY2638 | Derivative of NKY1551 with *dmc1::URA3* | This study |
| MSY4674 | Derivative of NKY1551 with *dmc1::URA3 xrs2–SH* | This study |
| MSY1817/2085 | Derivative of NKY1551 with *rad50S::URA3 xrs2–SH/xrs2–664* | This study |

**References**

1. Ma JL, Kim EM, Haber JE, Lee SE (2003) Yeast Mre11 and Rad1 proteins define a Ku-independent mechanism to repair double-strand breaks lacking overlapping end sequences. Mol Cell Biol 23: 8820-8828.

2. Matsuzaki K, Terasawa M, Iwasaki D, Higashide M, Shinohara M (2012) Cyclin-dependent kinase-dependent phosphorylation of Lif1 and Sae2 controls imprecise nonhomologous end joining accompanied by double-strand break resection. Genes Cells 17: 473-493.

3. Thomas BJ, Rothstein R (1989) Elevated recombination rates in transcriptionally active DNA. Cell 56: 619-630.

4. Matsuzaki K, Shinohara A, Shinohara M (2008) Forkhead-associated domain of yeast Xrs2, a homolog of human Nbs1, promotes nonhomologous end joining through interaction with a ligase IV partner protein, Lif1. Genetics 179: 213-225.

5. Storlazzi A, Xu L, Schwacha A, Kleckner N (1996) Synaptonemal complex (SC) component Zip1 plays a role in meiotic recombination independent of SC polymerization along the chromosomes. Proc Natl Acad Sci U S A 93: 9043-9048.

6. Shima H, Suzuki M, Shinohara M (2005) Isolation and characterization of novel xrs2 mutations in Saccharomyces cerevisiae. Genetics 170: 71-85.
