# Supplementary material for: The MRX Complex Ensures NHEJ Fidelity through Multiple Pathways Including Xrs2-FHA–Dependent Tel1 Activation
Source: PLoS Genet. 2016 Mar 18;12(3):e1005942. doi: 10.1371/journal.pgen.1005942 (PMC4798412; doi:10.1371/journal.pgen.1005942)
Supplement: S2 Table — (DOCX) [file pgen.1005942.s006.docx]

**Table S2.** **Percentage of each category of repaired products after induction of non-complementary DSBs**

|  | A | B | C | D | E | N^a^ |
| --- | --- | --- | --- | --- | --- | --- |
| Wild type | 33.3 | 20.9 | 3.1 | 7.0 | 37.2 | 258 |
| *xrs2-SH* | 31.8 | 20.6 | <0.58^b^ | 7.4 | 42.4 | 170 |
| *tel1-KN* | 0.8 | 17.9 | 2.4 | 11.4 | 67.5 | 125 |
| *xrs2-664* | 2.1 | 34.0 | 8.5 | 6.4 | 48.9 | 47 |
| *xrs2*∆ | 37.8 | 20.2 | 2.5 | 5.0 | 37.8 | 119 |
| *yku70*∆ | 95.4 | 4.5 | <4.5 ^b^ | <4.5 ^b^ | <4.5 ^b^ | 22 |

^a^N; Number of samples analyzed.

^b^No product was observed after indicated numbers of samples were analyzed.
