# Supplementary material for: The MRX Complex Ensures NHEJ Fidelity through Multiple Pathways Including Xrs2-FHA–Dependent Tel1 Activation
Source: PLoS Genet. 2016 Mar 18;12(3):e1005942. doi: 10.1371/journal.pgen.1005942 (PMC4798412; doi:10.1371/journal.pgen.1005942)
Supplement: S3 Table — (DOCX) [file pgen.1005942.s007.docx]

**Table S3. Relative frequencies of each category of repaired products after induction of non-complementary DSBs**

|  | Relative frequencies^a^ | A | B | C | D | E | Resection tract in class-B^b^ |
| --- | --- | --- | --- | --- | --- | --- | --- |
| Wild type | 1.00 | 0.33 | 0.21 | 0.03 | 0.07 | 0.38 | 21.7 |
| *xrs2-SH* | 2.41 | 0.64 | 0.57 | 0 | 0.11 | 1.08 | 17.9 |
| *tel1-KN* | 2.96 | 0.02 | 0.53 | 0.07 | 0.34 | 1.99 | 17.1 |
| *xrs2-664* | 4.60 | 0.10 | 1.57 | 0.39 | 0.29 | 2.25 | 17.8 |
| *xrs2*∆ | 0.22 | 0.08 | 0.04 | 0.01 | 0.01 | 0.08 | 3.0 |
| *yku70*∆ | 0.19 | 0.18 | 0.01 | 0 | 0 | 0 | N/A |

^a^Relative frequencies in each mutant cell compared to wild type was shown.

Average number of deleted nucleotide after repair.

^b^Average numbers of deleted nucleotides in the characterized products which were repaired by Ku-dependent imprecise end joining associated with end resection and microhomology (category B).

N/A; Not applicable
